# Using Surface Immunogenic Protein as a Carrier Protein to Elicit Protective Antibody to Multiple Serotypes for Candidate Group B Streptococcal Glycan Conjugate Vaccines

**DOI:** 10.3390/vaccines12060573

**Published:** 2024-05-24

**Authors:** Huiqi Duan, Wenhua Huang, Qingyu Lv, Peng Liu, Qian Li, Decong Kong, Xuyang Sun, Xinran Zhang, Yongqiang Jiang, Shaolong Chen

**Affiliations:** 1 School of Basic Medical Sciences, Anhui Medical University, Hefei 230032, China; 2 State Key Laboratory of Pathogen and Biosecurity, Academy of Military Medical Sciences, Beijing 100071, China; 3 Tianjin Key Laboratory of Agricultural Animal Breeding and Healthy Husbandry, College of Animal Science and Veterinary Medicine, Tianjin Agricultural University, Tianjin 300392, China

**Keywords:** *Group B Streptococcus* (GBS), surface immunogenic protein (Sip), glycan conjugate vaccine, serotype-independent protection

## Abstract

*Group B Streptococcus* (GBS) is a life-threatening opportunistic pathogen, particularly in pregnant women, infants, and the elderly. Currently, maternal vaccination is considered the most viable long-term option for preventing GBS mother-to-infant infection, and two polysaccharide conjugate vaccines utilizing CRM197 as a carrier protein have undergone clinical phase II trials. Surface immunogenic protein (Sip), present in all identified serotypes of GBS strains so far, is a protective surface protein of GBS. In this study, the type Ia capsular polysaccharide (CPS) of GBS was utilized as a model to develop candidate antigens for a polysaccharide conjugate vaccine by coupling it with the Sip of GBS and the traditional carrier protein CRM197. Serum analysis from immunized New Zealand rabbits and CD1 mice revealed that there was no significant difference in antibody titers between the Ia-Sip group and Ia-CRM197 group; however, both were significantly higher than those observed in the Ia polysaccharide group. Opsonophagocytosis and passive immune protection results using rabbit serum indicated no significant difference between the Ia-Sip and Ia-CRM197 groups, both outperforming the Ia polysaccharide group. Furthermore, serum from the Ia-Sip group had a cross-protective effect on multiple types of GBS strains. The challenge test results in CD1 mice demonstrated that the Ia-Sip group provided complete protection against lethal doses of bacteria and also showed cross-protection against type III strain. Our study demonstrates for the first time that Ia-Sip is immunogenic and provides serotype-independent protection in glycan conjugate vaccines, which also indicates Sip may serve as an excellent carrier protein for GBS glycan conjugate vaccines and provide cross-protection against multiple GBS strains.

## 1. Introduction

*Group B Streptococcus* (GBS), also known as *Streptococcus agalactiae*, is a life-threatening opportunistic pathogen, particularly in pregnant women, infants, and the elderly [1,2]. It is estimated that 15% to 25% of pregnant women worldwide (approximately 20 million) are colonized with this pathogen in their reproductive tract annually, with 50% transmitting it vertically to their offspring without intervention [1,2,3]. GBS infections are responsible for an estimated 46,000 stillbirths and 91,000 newborn deaths each year. GBS is the leading cause of neonatal morbidity and mortality, accounting for over half of neonatal infections within the first three months of life [1,2,4]. This includes early onset infection acquired through the maternal birth canal within 7 days after birth and late onset infection acquired from either the mother or the environment between 7 and 89 days after birth [4]. Typically causing neonatal sepsis and meningitis, GBS results in irreversible neurological damage such as cerebral palsy, hearing loss, and vision impairment affecting approximately 37,000 infants annually, with a case fatality rate of approximately 5% [1,2,4,5]. Furthermore, there has been a more than twofold increase in aggressive GBS cases among older adults (≥65 years) over the past two decades, especially those with comorbidities like diabetes and obesity, with a case fatality rate ranging from 10 to 15% [2,6,7,8,9].

The current primary clinical approach to reducing invasive GBS infection is intrapartum antimicrobial prophylaxis (IAP), involving the pre-administration of penicillin and macrolide antibiotics to pregnant women with GBS pathogens after being screened [5,10,11,12]. However, IAP only reduces early-onset disease (EOD) infections by more than 80% and is ineffective against late-onset disease (LOD) [5,10,13]. Additionally, there is an increasing resistance in GBS strains, and the use of antibiotics can disrupt the establishment of normal flora in newborns, impacting their normal development [5,10]. Maternal immunization with a GBS vaccine is considered a superior prevention strategy as it not only addresses the remaining burden of EOD and LOD but also prevents the stillbirths and premature births caused by GBS during pregnancy while avoiding early infant susceptibility to GBS threats [11,14,15]. Moreover, it reduces reliance on prophylactic antibiotics and minimizes antibiotic resistance concerns [14,15]. Currently, maternal vaccination is the most viable long-term option for preventing the mother-to-child transmission of GBS infection and has been prioritized by the WHO, CDC, UK Department of Public Health, and the Bill & Melinda Gates Foundation [16,17,18,19,20].

Since Lancefield et al. first demonstrated in 1966 that the capsular polysaccharide (CPS) of GBS can elicit a humoral immune response in mice, generating serotype-specific antibodies against fatal GBS infection in a mouse model, the initial generation of GBS vaccine was developed based on purified natural CPS antigen [21,22,23,24,25]. The CPS of GBS mainly includes 10 serotypes, including Ia, Ib, II, III, IV, V, VI, VII, VIII, and IX. Among them, serotypes Ia-V account for more than 97% of clinical isolates worldwide, while in China, serotypes Ia, Ib, III, and V are responsible for 98% of invasive GBS infections [1,2,3,26]. Polyvalent CPS vaccines have demonstrated favorable safety and immunogenicity profiles: vaccinated individuals either exhibit no reaction or only mild local reactions; the serum from immunized individuals exhibits opsonophagocytic activity against GBS and is capable of protecting animals from lethal doses in animal models with GBS infection [27]. However, this polysaccharide vaccine has low immunogenicity as a non T-cell dependent antigen; the level of immunoglobulin produced by this vaccine is lower than that generated by vaccines designed later and it lacks immune memory function [28,29]. Moreover, most maternal antibodies are unable to cross the placenta, thus providing only short-term protection to the fetus without conferring distinct protective effects on newborns postpartum [30].

Therefore, following the first U.S. approval of the Hemophilus influenzae capsular polysaccharide–diphtheria toxoid conjugate vaccine in 1990 and its excellent results [31], conjugate vaccines that are conjugated with the CPS of GBS and Tetanus Toxoid (TT) or CRM197 (cross-reacting material 197, a detoxified form of diphtheria toxin) represent the second-generation vaccines for GBS [32]. Upon the covalent coupling of polysaccharide and protein, this vaccine enters B cells through B cell receptors, where the conjugate protein carrier presents antigen epitopes to T cells in peptide form through the major histocompatibility complex (MHC) on B cells, thereby promoting the maturation and proliferation of plasma cells (which can produce polysaccharide-specific antigens) as well as the generation of memory B cells [28,33,34,35]. This process leads to a stronger and more functional IgG response through antibody class switching. Therefore, coupling with a carrier protein effectively addresses the issue of low immunogenicity and the inability to induce the immune memory associated with polysaccharide vaccines [21,28,29,33,35]. This approach can directly protect mothers and newborns through maternal immunity (antibodies can reach newborns via the placenta and persist for over 2 months), while also reducing the risk of LOD in newborns [21,22,23,27]. Currently, Pfizer’s hexavalent capsular polysaccharide-CRM197 conjugate vaccine has completed phase II clinical trials, demonstrating good tolerability and strong maternal antibody response that can be effectively transferred to infants for protection against invasive GBS [29,36]. Similarly, Glaxo Smith Kline (GSK)’s trivalent capsular-CRM197 conjugate vaccine showed significantly higher levels of specific antibodies in both maternal and infant serum, as well as secretory antibodies in maternal milk compared to the control group during a 90-day postpartum period [37,38,39]. However, it is worth noting that, as the vector of the already marketed *Hemophilus influenzae*, *Neisseria meningitidis* and *Streptococcus pneumoniae* polysaccharide-conjugate vaccine, CRM197, may lead to carrier-induced epitopes suppression (CIES), resulting in carrier-induced immunosuppression, which could reduce immune responses against polysaccharide antigens [28,34]. Like the polysaccharide vaccine, polysaccharide conjugate vaccine is needed to develop polyvalent vaccine to maximize the coverage of clinical GBS pathogenic strains.

Furthermore, protein subunit vaccines targeting GBS surface proteins, such as Alpha C protein, Rib protein, surface immunogenic protein (Sip), Beta C protein, C5a peptidase (ScpB), Srr1, BibA, GBS80, and their fusion proteins, have been explored [40,41,42,43]. These protein vaccines demonstrate good immunogenicity and are capable of inducing specific Ig antibodies in mice. However, it is critical to note that a single protein typically fails to cover all clinical isolate serotypes. Currently, Minervax has successfully developed a phase I clinical-trial-ready protein vaccine (GBS-NN) based on the N-terminal fusion domain of Alpha C and Rib with promising results in terms of tolerability and high immunogenicity, showing excellent application prospects [44,45].

By utilizing the surface protein of GBS as a carrier protein instead of TT or CRM197 to induce a T-cell dependent immune response, not only can the attenuated immune response to the polysaccharide antigen caused by CIES be avoided, but also the highly conserved nature of the GBS surface protein can broaden the vaccine’s protective range against various serotypes of GBS infection, thus enabling it to provide multiple protections. In this study, we used monovalent type Ia CPS as an example and developed a candidate antigen for glycan conjugate vaccine through coupling with the Sip of GBS and traditional carrier CRM197; the Ia-Sip group showed similar or better results than the Ia-CRM197 group in antibody titers, opsonophagocytosis, and passive immune protection experiments. Furthermore, serum from the Ia-Sip group had a cross-protective effect on multiple types of GBS strains. Our findings suggest for the first time that Ia-Sip exhibits strong immunogenicity and enables glycan conjugate vaccines to provide serotype-independent protection, which also indicates Sip’s potential as an outstanding carrier protein.

## 2. Materials and Methods

### 2.1. Strain Information and Culture Conditions

In this study, the GBS strains utilized were as follows: Ia strain 515 (ATCC (BAA-1177)), a strain isolated from neonatal cerebrospinal fluid; Ib strain SA-1, a clinical isolate from neonatal cerebrospinal fluid provided by Children’s Hospital of Capital Medical University (56 Nanlishi Lu, Xicheng District, Beijing, China); II strain SA-28, also obtained from a clinical isolate provided by Children’s Hospital of Capital Medical University; III strain COH1 (ATCC (BAA-1176)), derived from patient blood; and V strain 2603V/R (ATCC (BAA-611)), a clinical isolate. All ATCC standard strains were maintained in our laboratory.

The bacterial strains were cultured on Colombia agar plates (BD, Franklin Lakes, NJ, USA) supplemented with 5% sheep’s blood (Solarbio, Beijing, China) at 37 °C under a 5% CO_2_ atmosphere. The bacterial suspensions were incubated in Todd Hewitt broth (THB, BD, Franklin Lakes, NJ, USA) without agitation for 4.5 h and subsequently centrifuged to adjust the OD600 to 0.3 for subsequent experiments including opsonophagocytosis and passive immune protection.

### 2.2. Purification of Capsular Polysaccharides Type Ia

The capsular polysaccharides type Ia was purified using a combination of established methods [46,47] and an innovative anion chromatography technique. Specifically, the glycerol-frozen strain Ia 515 was revived from −80 °C and cultured in Columbia medium (BD, Franklin Lakes, NJ, USA) before being transferred to fermentation seed solution for 5 h. Fermentation was then carried out in an 8 L fermenter with Columbia medium (BD, Franklin Lakes, NJ, USA) supplemented with 8% glucose (Sinopharm Chemical Reagent Co., Ltd., Shanghai, China) for 16 h while maintaining pH at 7 and aeration rate at 5 L/min. The resulting broth was harvested by centrifugation and filtration, followed by two rounds of overnight precipitation with 25% and 75% ethanol (Sinopharm Chemical Reagent Co., Ltd., Shanghai, China) to obtain crude sugars. These crude sugars were further separated using N, N-Diethylethanolamine (DEAE) Sepharose Fast Flow chromatography (Cytiva, Shanghai, China), with the purified polysaccharides collected at the peak corresponding to UV absorption at 206 nm. Finally, ultrafiltration with water for injection yielded highly pure capsular polysaccharides that underwent size and structure analysis.

### 2.3. Quantification and Verification of Type Ia CPS

The content of the purified CPS was determined by the phenol-sulfuric acid method, while glucose served as the standard. After weighing and drying in an oven at 56 °C, glucose was dissolved in ultrapure water. Refined CPS samples were treated with concentrated sulfuric acid (Sinopharm Chemical Reagent Co., Ltd., Shanghai, China) and 5% phenol (Solarbio, Beijing, China), followed by incubation in a water bath at 80 °C for 20 min. The absorbance was measured at OD485 nm to calculate the content of CPS. This method involved hydrolyzing the polysaccharide into monosaccharides through concentrated sulfuric acid action and dehydrating to form sugar aldehyde derivatives that react with phenol resulting in an absorption peak at 485 nm.

Furthermore, the protein content was determined using the bicinchoninic acid (BCA) method, while the nucleic acid content was quantified via ultraviolet spectrophotometry.

### 2.4. Molecular Weight and Structure Identification of Type Ia CPS

Molecular weight determinations of type Ia CPS were conducted using high-performance liquid chromatography (HPLC, waters, Milford, MA, USA) coupled with a differential detector. The HPLC system employed OHpak SB-804 HQ and OHpak SB-805 HQ liquid chromatographic columns (Shodex, Nagoya, Japan), while the mobile phase consisted of a 150 mM phosphate buffer solution at pH 6.86. Detection was carried out using a differential detector. A standard curve was established using STANDARD P-82 (F8400000, Shodex, Nagoya, Japan), which was prepared by dissolving it in the mobile phase to achieve a concentration of 2 mg/mL.

The purified CPS was lyophilized and subjected to Nuclear Magnetic Resonance (NMR) H spectrum analysis for structural identification.

### 2.5. Construction and Purification of Recombinant Sip Protein

Following established protocols [48,49], the sequence encoding Sip without signal peptides region (27 aa–434 aa) was successfully inserted into the pET28a plasmid DNA to generate the pET28a::*sip* gene construct. Subsequently, this plasmid was efficiently transformed into *E. coli* BL21(DE3) competent cells to enable robust expression of recombinant Sip protein. The resulting protein product was then purified using a Ni column affinity chromatography (Cytiva, Shanghai, China) technique. To assess its quality, sodium dodecyl sulfate–polyacrylamide gel electrophoresis (SDS-PAGE) analysis was performed and quantification was carried out using the BCA method.

### 2.6. Preparation of Immune Antigens as Candidate Glycan Conjugate Vaccines

**Figure 2:**
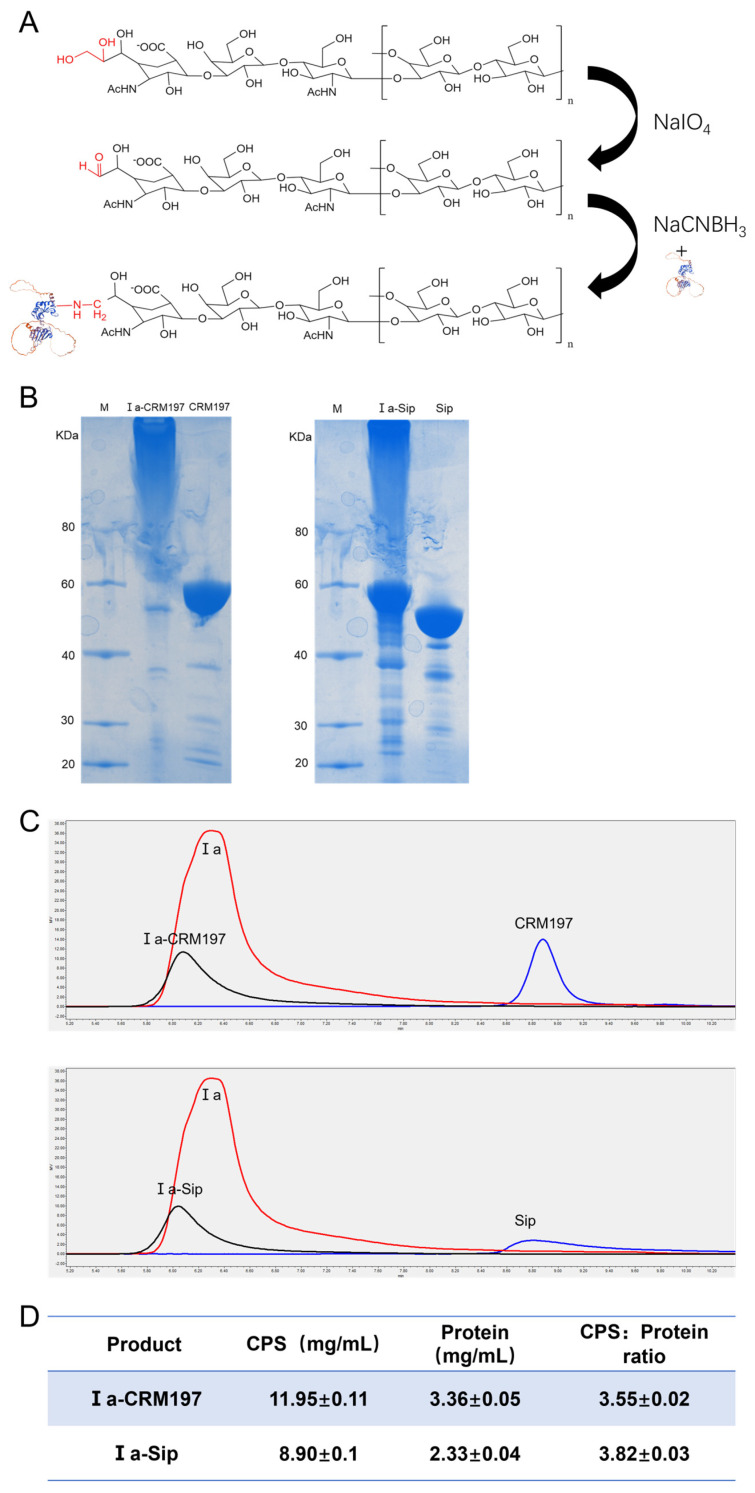
Confirmation of type Ia glycan conjugate vaccine. (**A**) Schematic model of the coupling process of type Ia CPS with traditional carrier protein CRM197 and *Group B Streptococcus* (GBS) surface protein Sip. (**B**) The SDS-PAGE gel electrophoresis of the coupling conjugate with the original protein. (**C**) The HPLC analysis of the coupling conjugate with the original protein and type Ia CPS. (**D**) The content of CPS in the coupling conjugate was determined by the phenol-sulfuric acid method, while the protein was quantified using the BCA method. Then, the ratio of polysaccharide to protein was calculated for both coupling products. Data are presented as means ± SD.

The candidate antigens of Ia polysaccharide conjugate vaccine were prepared as shown in Figure 2A. The purified Ia CPS was coupled with the traditional carrier CRM197 and GBS surface protein Sip, respectively. 2 mg/mL of polysaccharide (Sinopharm Chemical Reagent Co., Ltd., Shanghai, China) dissolved in a 50 mm sodium acetate buffer solution (pH 4.5) was activated with sodium periodate (Macklin, Shanghai, China) for 2 h, and then the reaction was terminated with ethylene glycol (Sinopharm Chemical Reagent Co., Ltd. Shanghai, China). The activated CPS was conjugated to the two proteins at a ratio of 4:1 using sodium cyanoborohydride (Sigma, Darmstadt, Germany), followed by termination of the reaction with 2 mg/mL sodium borohydride (Sigma, Darmstadt, Germany). The capsular polysaccharide-conjugate antigen was obtained through ultrafiltration using water for injection. SDS-PAGE analysis was performed and quantification was carried out using the phenol-sulfuric acid method and the BCA method as described above.

### 2.7. Animal Experiments and Challenge

**Figure 3:**
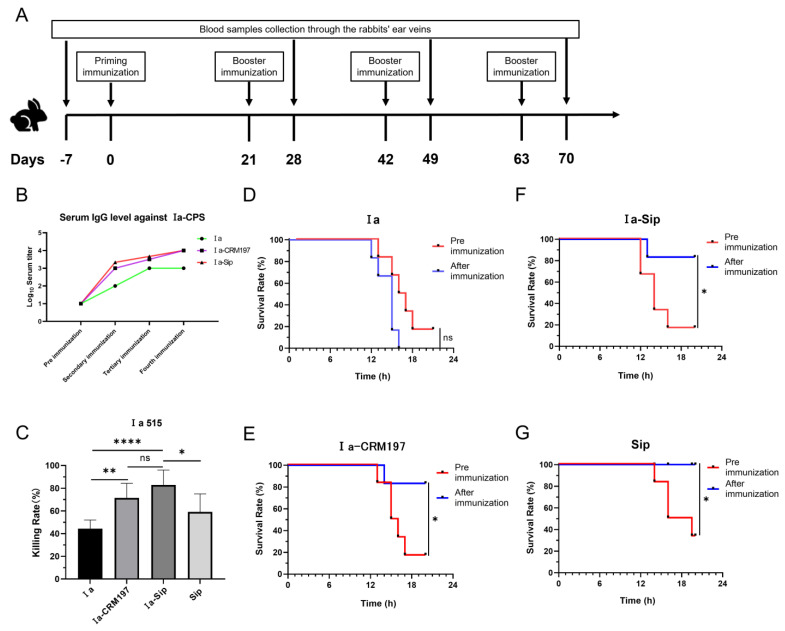
Rabbit serum titers after immunization and its protective efficacy against serotype Ia *Group B Streptococcus*. (**A**) The immunization and blood sample collection process of New Zealand white rabbits. (**B**) Rabbit serum IgG titers against type Ia CPS were determined by ELISA; data represent mean values of two or three rabbit serum titers according to the rabbits used. (**C**) The opsonophagocytic assay using rabbit sera after the third immunization with differentiated HL60 cells against serotype Ia GBS strains; data are presented as means ± SD of at least four individual experiments. Unpaired Student *t*-tests were used for statistical analysis. (* *p* < 0.05, ** *p* < 0.01, **** *p* < 0.0001 and ns represents no significance). (**D**–**G**) The passive immune protection experiments against serotype Ia GBS strains were performed on mice using rabbit sera after the fourth immunization, and the equivalent dose of corresponding rabbit sera before immunization was used as the control. Survival curves of mice protected by rabbit serum from Ia group (**D**), Ia-CRM197 group (**E**), Ia-Sip group (**F**), and Sip group (**G**) are presented here. (* *p* < 0.05, and ns, no significance).

New Zealand white rabbits weighing 3 kg were immunized to obtain immune serum, as shown in Figure 3A. The rabbits were immunized intramuscularly with Ia CPS (50 μg per rabbit), Ia-CRM197 (50 μg Ia per rabbit), Ia-Sip (50 μg Ia per rabbit), and recombinant Sip protein (50 μg per rabbit) at 0, 21, 42, and 63 days after mixing with Freund’s adjuvant (Sigma, Darmstadt, Germany) (Freund’s complete adjuvant for the priming immunization and Freund’s incomplete adjuvant for the booster immunization), respectively. Blood was collected from the ear vein seven days before immunization and seven days after each booster immunization for serum separation.

Sixty eight-week-old female CD1 mice were randomly divided into five experimental groups with twelve mice in each group. Mice were immunized intraperitoneally three times at 0, 21, and 42 days with Ia CPS (5 μg per mouse), Ia-CRM197 (5 μg Ia per mouse), Ia-Sip (5 μg Ia per mouse), and recombinant Sip protein (5 μg per mouse) after mixing with Freund’s adjuvant (Freund’s complete adjuvant for the priming immunization and Freund’s incomplete adjuvant for the booster immunization). Additionally, blood was collected from the tail vein of CD1 mice seven days before immunization, seven days after the secondary and tertiary vaccination for serum separation. Ten days after the third immunization, the survival of the mice was assessed using a dose of 3.50 × 10^8^ bacteria of strain Ia 515 intraperitoneally. For challenge experiments to verify cross-protection using type III strains, four groups consisting of 48 mice in total were prepared (excluding the group that received with Ia CPS immunization). The mice were challenged intraperitoneally with COH1 strain at a dose of 6.85 × 10^8^ bacteria ten days post triple-immunizations.

### 2.8. ELISA for the Determination of Antibody Titers in Rabbit and Mouse Sera

An enzyme-linked immunosorbent assay (ELISA) was employed to determine the titer of type Ia CPS in rabbit and mouse sera, as well as the titer of type-specific antibodies in mouse sera. Specifically, ELISA plates were coated with Ia CPS (1 μg/mL for rabbits and 3 μg/mL for mice) at 4 °C overnight and subsequently blocked with 3% Bovine Serum Albumin (BSA) (Coolaber, Beijing, China) overnight at 4 °C. Serial dilutions of sera were then incubated at 37 °C for 30 min, followed by incubation with Horseradish peroxidase (HRP)-labeled goat anti-rabbit IgG and goat anti-mouse IgG secondary antibodies (1:1000 dilution, TransGen, Beijing, China) at 37 °C for 30 min. The reaction was developed using substrate 3,3′,5,5′-Tetramethylbenzidine (TMB) (Sigma, Darmstadt, Germany) and terminated with sulfuric acid (1 M), after which the absorbance at a wavelength of 450 nm was measured using a microplate reader (Molecular Devices, San Jose, CA, USA). For determining the class of typing antibodies, HRP-labeled goat anti-mouse IgG1, Ig2a, Ig2b, Ig3, IgM, and IgA (Abcam, Cambridge, UK) were employed as secondary antibodies.

### 2.9. Opsonophagocytic Assay

The opsonophagocytic assay was conducted following established protocols in the literature [50,51,52]. Specifically, HL60 cells were cultured and passaged in Iscove’s Modified Dulbecco Medium (IMDM) (Pricella, Wuhan, China) supplemented with 20% Fetal Bovine Serum (FBS) (Gibco, New York, NY, USA) and 1% penicillin-streptomycin at 37 °C under a 5% CO_2_ atmosphere. The differentiation of HL60 cells into neutrophils was induced using 1% sterile Dimethyl sulfoxide (DMSO) (Sigma, Darmstadt, Germany), followed by culturing in IMDM medium containing 10% serum for 2–3 days. Flow cytometry analysis revealed approximately 85% of the cells successfully differentiated, as evidenced by CD11b (BioLegend, San Diego, CA, USA) expression. Phagocytosis assays were performed on microplates (Corning, Allegan, MI, USA) by incubating bacterial solutions of 5 × 10^3^ CFU per well mixed with 10 μL inactivated triple immunized rabbit serum at 37 °C for 30 min, followed by the addition of 10 μL neonatal rabbit complement (ReI·Freez) and activated HL60 cells at a concentration of 5 × 10^6^ per well, which were then incubated for an additional 30 min. After the completion of the incubation period, colony-forming units (CFUs) were counted on agar plates to determine bactericidal activity. Pre-immunized serum from the same rabbit served as a control to calculate the bactericidal rate under the same experimental conditions.

### 2.10. Passive Immune Protection of Rabbit Serum in Mice

The passive immune protection experiments were conducted in accordance with the previous literature [53]. Six four-week-old female CD1 mice received intraperitoneal injections of 40 μL of rabbit serum after four immunizations 12 h before the challenge. As a control, an equivalent dose of corresponding rabbit serum before immunization was injected into 6 mice in the control group. The Ia 515 strain was then intraperitoneally injected at a dosage of 1.15 × 10^5^ CFU per mouse for subsequent observation of mice survival. To evaluate cross-protection against the type Ib strain (SA-1)/the type III strain (COH1)/the type V strain (2603V/R), mice in the experimental group also received injections of immunized rabbit serum from either the Ia-Sip or Sip groups, while the control group received a mixture containing equal proportions of both sera before immunization and were subsequently challenged with a dosage of 7.9 × 10^5^ CFU/1.33 × 10^5^ CFU/5.75 × 10^5^ CFU per mouse via intraperitoneal injection.

### 2.11. Ethics Statement

All experimental procedures involving mice and rabbits were conducted in strict accordance with the guidelines established by the Chinese Regulations of Laboratory Animals and Laboratory Animal Requirements of Environment and Housing Facilities. These procedures were approved by the Institutional Animal Care and Use Committee of the AMMS under permit no. IACUC of DWZX-2023-051 and DWZX-2024-005, ensuring compliance with ethical standards. Consideration was given to animal welfare, with efforts made to minimize any potential suffering.

### 2.12. Statistical Analyses

The results are presented as the mean and standard deviation in a bar graph. The statistical data analysis was performed using the *unpaired t-test* and *ANOVA test*, and *p*-values < 0.05 were considered statistically significant. *Log-rank (Mantel-Cox) test* was used for analyses of survival curves, and results with 95% confidence intervals were accepted. The analyses were performed using GraphPad Prism software 8.0.2.

## 3. Results

### 3.1. The Purified Type Ia CPS Exhibits High Purity, Accurate Size, and Proper Structure

**Figure 1:**
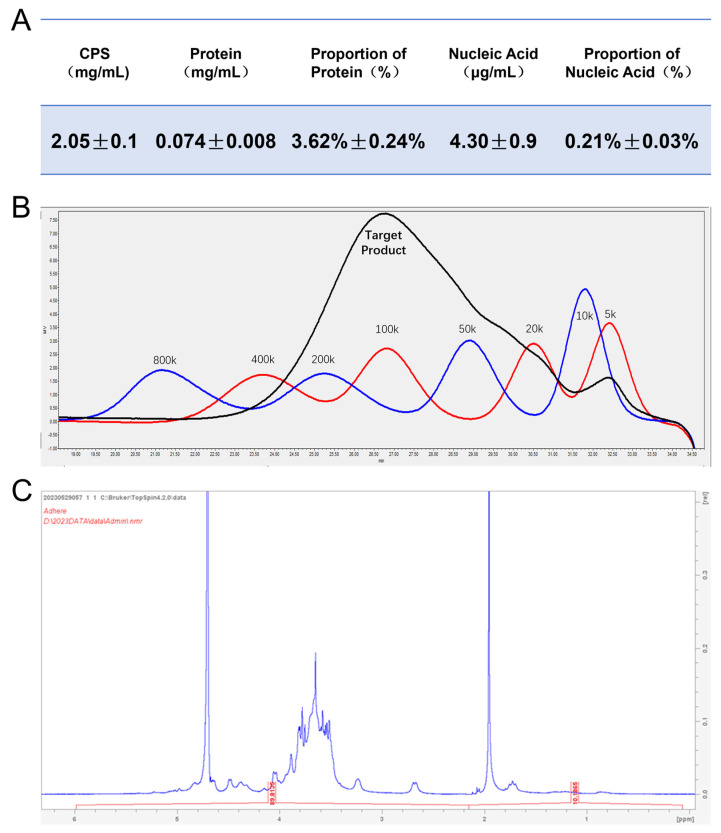
Identification of serotype Ia capsular polysaccharide (CPS). (**A**) The content of the purified CPS was quantified using the phenol-sulfuric acid method, while the protein and nucleic acid contents were measured via the BCA method and ultraviolet spectrophotometry. Then, the protein and nucleic acid impurities in purified type Ia CPS were calculated. Data are presented as means ± SD. (**B**) The molecular weight of purified type Ia CPS was determined by HPLC coupled with a differential detector, using dextran as a standard reference. (**C**) The NMR H spectrum analysis was conducted on freeze-dried purified type Ia CPS.

We quantified the levels of CPS, protein impurities, and nucleic acid impurities in the purified type Ia CPS, as depicted in Figure 1A. The measured protein and nucleic acid impurities were approximately 3.62% and 0.21%, respectively, indicating a low level of impurities in the obtained capsular polysaccharides. Then, we determined the molecular weight of purified CPS using HPLC (Figure 1B). By employing dextran as a standard reference, the molecular weight of purified type Ia CPS was approximately 100 kDa. Following lyophilization, NMR H-spectrum analysis confirmed that characteristic peaks in the samples were completely consistent with values reported in the literature [40,54] and no significant miscellaneous peaks were observed (Figure 1C). Based on these findings, it can be concluded that highly pure serotype Ia CPS from GBS strain 515 have been successfully obtained with a consistent size and structure, as reported in previous studies.

### 3.2. Type Ia CPS Was Successfully Coupled with Carrier Protein CRM197 and GBS Surface Protein Sip

The process of coupling type Ia CPS with the traditional carrier proteins CRM197 and GBS surface protein Sip is illustrated in Figure 2A. The cis-o-dihydroxyl group on the side chain of type Ia CPS is oxidized to an active aldehyde group by sodium iodate, which then forms an unstable intermediate when covalently bound to the two proteins in solution at the appropriate ratio. This intermediate can be further reduced with sodium cyanoborohydride to yield a stable conjugated product. SDS-PAGE analysis revealed that, compared to the original proteins, the band corresponding to the conjugated products appeared more concentrated at the loading port. This suggests that the conjugated product had a larger molecular weight and potentially formed a complex spatial structure, preventing it from entering into the SDS-PAGE gel (Figure 2B). Additionally, HPLC detection showed that the retention time of the conjugate on chromatography was significantly shorter than that of Ia polysaccharide or protein alone, with a single peak type observed (Figure 2C), suggesting the successful formation of the conjugate. Finally, we determined that, in both coupling products, Ia-CRM197 had a polysaccharide-to-protein ratio of 3.56 and Ia-Sip had a ratio of 3.82 (Figure 2D).

### 3.3. The Serum IgG Antibody Level of Rabbits Was Higher When Immunized with Glycan Conjugate Vaccine Than When Immunized with Capsular Polysaccharide Vaccine

The immunization process of New Zealand white rabbits is depicted in Figure 3A. The serum titers of the Ia, Ia-CRM197, and Ia-Sip groups were determined by indirect ELISA using type Ia CPS antigen. Interestingly, it was observed that, following the second round of immunization, the CPS serum titer of the polysaccharide conjugate vaccine as an antigen was significantly higher than that of CPS alone (Figure 3B), consistent with previous reports indicating a lower IgG level generated by animals stimulated with polysaccharide antigens than those stimulated with polysaccharide conjugate product [21,55,56].

### 3.4. Rabbit Serum from the Ia-Sip Group and the Ia-CRM197 Group Exhibited Comparable Protective Effects against Type Ia 515 GBS Strains in Both HL60 Opsonophagocytosis Test and Passive Immune Protection Experiments in Mice

Antibodies against CPS in rabbit serum were quantitatively measured by ELISA, while neutralizing antibodies against GBS type Ia strain were evaluated through an HL60 opsonophagocytosis assay and passive immune protection assay in mice. HL60 cells were induced to differentiate into neutrophils by DMSO, and the phagocytosis and sterilization of the GBS type Ia strain in the presence of rabbit serum were tested. The results depicted in Figure 3C indicate that the bactericidal rates of the Ia-CRM197 (71.39 ± 12.88%) and Ia-Sip (82.98 ± 13.02%) groups were significantly higher than that of the Ia group (44.41 ± 7.66%) (**, *p* = 0.0011 and ****, *p* < 0.0001). However, there was no significant difference between these two groups (*p* = 0.0683), with only a slightly higher bactericidal rate observed in the Ia-Sip group. Furthermore, the bactericidal rate of the Ia-Sip group was also higher than that of Sip protein alone (59.12 ± 15.87%) (*, *p* = 0.0127). In this experiment, the rabbit serum of the corresponding rabbit before immunization was used as a control to calculate the bactericidal rate to exclude the interference of serum background. On the other hand, we confirmed the protective effect of rabbit serum at the animal level through passive immune protection experiments on young mice (Figure 3D–G). Pre- and post-immunization rabbit sera were intraperitoneally injected into mice, followed by challenges with strain Ia 515 after 6 h. The sera from the immunized rabbits in the Ia group did not protect against lethal doses of Ia strain infection (five out of six mice died using pre-immunization serum vs. all six mice died using post-immunization serum, Figure 3D). In contrast, the Ia-CRM197 group (five out of six mice died using pre-immunization serum vs. one out of six mice died using post-immunization serum, Figure 3E), Ia-Sip group (five of six died using pre-immunization serum vs. one of six died using post-immunization serum, Figure 3F), and Sip group (four of six died using pre-immunization serum vs. all six survived using post-immunization serum, Figure 3G) all showed excellent protective effects. From these findings, it is evident that the Ia-Sip antigen we constructed in this study confers comparable protective effects against GBS type Ia strain at both the cellular and animal levels when compared to the traditional polysaccharide conjugate vaccine antigen Ia-CRM197.

### 3.5. Rabbit Serum from Ia-Sip Group Had Cross-Protective Effects against Multiple Serotypes of GBS Strains

**Figure 4:**
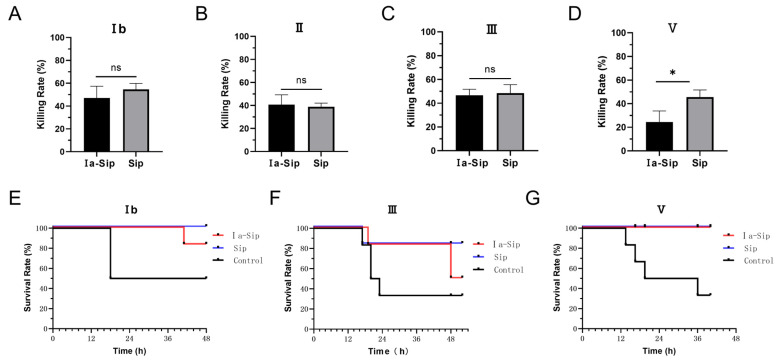
Protective efficacy against multi-serotype *Group B* *Streptococci* by immunized rabbit serum. (**A**–**D**) The opsonophagocytic assay was conducted using rabbit sera after the third immunization and differentiated HL60 cells. Different serotypes of GBS strains, including Ib strain SA-1 (**A**), II strain SA-28 (**B**), III strain COH1 (**C**), and V strain 2603V/R (**D**), were utilized and data are presented as means ± SD of at least three individual experiments. Unpaired Student *t*-tests were used for statistical analysis. * *p* < 0.05, and ns indicates no significance. (**E**,**F**) The passive immune protection experiments were performed on mice using rabbit sera after the fourth immunization from the Ia-Sip group and Sip group, with the control group receiving a mixture containing equal proportions of both sera before immunization. Survival curves of mice challenged by Ib strain SA-1 (**E**), III strain COH1 (**F**), and V strain 2603V/R (**G**) are shown here.

Due to the high conservation of Sip protein across different GBS strains and its confirmed immune protective effect, we hypothesized that coupling type Ia CPS with Sip could confer cross-protective effects against multiple serotypes of GBS strains. Our experimental results validated this hypothesis: At the cellular level, serum from the Ia-Sip group exhibited approximately 40% bactericidal activity against GBS strains of different serotypes (Figure 4A–D; Ib 47.05 ± 10.28%, II 40.70 ± 8.55%, III 46.73 ± 4.99%, V 24.50 ± 9.32%), which was significantly lower than that of type Ia at 82.98 ± 13.02%. However, the opsonophagocytosis effect was similar to that of Sip group serum (no statistical difference for type II, III, IV; *p* = 0.0139 for type V). At the animal level, the Ia-Sip group demonstrated a certain degree of protection against different GBS strain types (Figure 4E–G). Specifically, for type Ib GBS strains, survival rates were higher in the Ia-Sip group (five out of six animals) compared to the control group (three out of six animals); for type III GBS strains, more animals survived in the Ia-Sip group (three out of six animals) compared to control group (two out of six animals); and all animals survived in the Ia-Sip group when challenged with type V GBS strains, as opposed to only two surviving of the control groups’ six subjects each time tested. These results demonstrate that the constructed Ia-Sip antigen exhibits cross-protective effects against multiple serotypes of GBS strains.

### 3.6. The Mice Immunized with Ia-Sip Demonstrated Resistance to a Lethal Dose of Type Ia GBS Strain Challenge and Also Exhibited Cross-Protection against Type III GBS Strain

**Figure 5:**
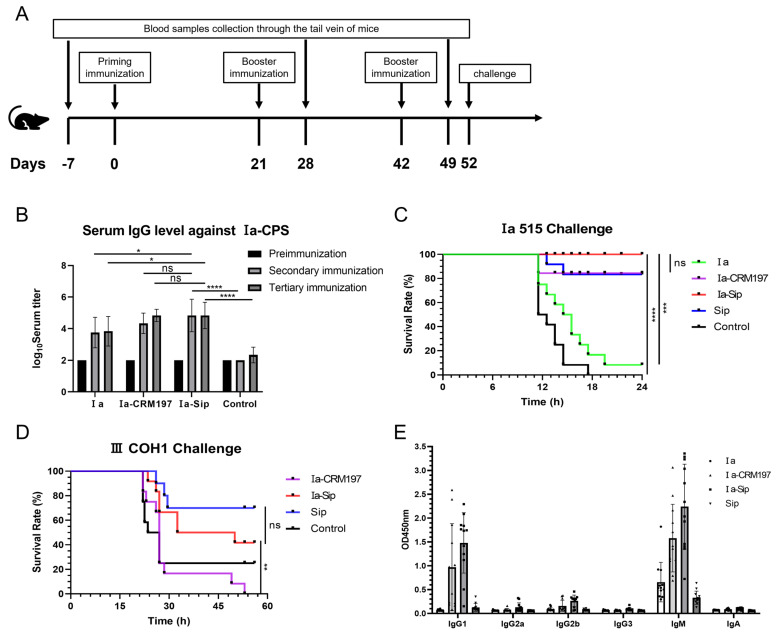
Mice serum titers and challenge against type Ia and III *Group B Streptococci.* (**A**) The immunization process and challenge experiments of CD1 mice. (**B**) Mice serum IgG titers against type Ia CPS were determined by ELISA; the figure is presented as means ± SD. (**C**) Survival curves of mice challenged by serotype Ia GBS strain 515 at a dose of 3.5 × 10^8^ CFU per mouse with 12 mice in each group are presented here. The CD1 mice were immunized three times using PBS (Control), Ia, Ia-CRM197, Ia-Sip, and Sip, each premixed with Freund’s adjuvant before challenge. (**D**) Survival curves of mice challenged by serotype III GBS strain COH1 at a dose of 6.8 × 10^8^ CFU per mouse with 12 mice in each group are shown here. The CD1 mice were immunized three times using PBS (Control), Ia-CRM197, Ia-Sip, and Sip, each premixed with Freund’s adjuvant before challenge. (**E**) The class of typing antibody titers in mouse serum against type Ia CPS were determined by ELISA, and data are presented as means ± SD of all the 12 immunized animals. * *p* < 0.05, ** *p* < 0.01, *** *p* < 0.001, **** *p* < 0.0001 and ns represents no significance.

The immunization process of CD1 mice and the challenge point are illustrated in Figure 5A. Consistent with the rabbit immunization results, the CPS serum titer in mice with the polysaccharide conjugate vaccine as antigen was significantly higher than that using the CPS alone as antigen in two measurements after the second immunization (Figure 5B). Subsequently challenged intraperitoneally with strain Ia 515 at a dose of 3.5 × 10^8^ CFU per mouse, all mice in the control group vaccinated with PBS succumbed to infection, while all mice in the Ia-Sip group survived without any adverse reactions such as mental malaise (Figure 5C). Only one mouse in the Ia polysaccharide group survived but exhibited signs of distress, while ten mice in the Ia-CRM197 or Sip groups also survived. To assess the cross-protection conferred by Ia-Sip group, a similar immunization excluding the Ia CPS group was performed followed by an intraperitoneal challenge experiment using 6.8 × 10^8^ CFU type III COH1 strains. All 12 mice in the Ia-CRM197 group succumbed to infection, whereas 2 mice in the control group and 5 mice in the Ia-Sip group survived (Figure 5D), indicating partial cross-immune protection effect derived from Sip protein as a carrier, consistent with our expectations. Based on these findings, it can be concluded that mice vaccinated with Ia-Sip demonstrated resistance against lethal doses of type Ia GBS strains and exhibited cross-protection against type III strains.

### 3.7. Compared with the Ia CPS Group, the Ia-Sip Group Induced Higher Levels of IgM Antibodies and Significantly Increased the Levels of IgG1 and IgG2a Antibodies

The traditional CPS is T lymphocyte-independent antigens, predominantly producing IgM antibodies that cannot cross the placenta, thereby primarily protecting against GBS infection in the mother. Our experimental results also support this conclusion (Figure 5E). However, conjugating CPS with the traditional carrier CRM197 and GBS surface protein Sip enables antigen epitope transfer to T cells, triggering cellular immunity and generating IgG antibodies that can cross the placenta. After being conjugated with the carrier protein, both the Ia-CRM197 group and Ia-Sip group exhibited significantly higher levels of IgM antibodies compared to the Ia group, while also generating a substantial amount of IgG1 and a minor proportion of IgG2a antibodies. This indicates that the conjugate vaccine induces both Th1 and Th2 immune responses, facilitating immune memory formation and conferring protection to newborns via transplacental transfer. Additionally, Ⅰa-Sip slightly enhances IgA expression levels which aid in acquiring relevant GBS-specific antibodies from maternal milk for defense resist late-onset GBS infection.Furthermore, under identical experimental conditions, the average antibody titers produced by the Ia-Sip group surpassed those generated by the Ia-CRM197 group.

## 4. Discussion

Currently, GBS represents the most significant infectious risk during pregnancy. The substantial burden of GBS disease and the unmet medical needs require an urgent focus on targeted prevention and treatment strategies for GBS [14,18]. To date, two polysaccharide conjugate vaccines and one fusion protein subunit vaccine have undergone clinical phase II trials, demonstrating favorable safety and immunogenicity profiles. Trivalent (Ia, Ib, III) vaccines from GSK and hexavalent (Ia, Ib, II-V) vaccines from Pfizer can provide coverage for over 90% of clinical GBS isolates [29,36,37,38,39]. Similar coverage is achieved with fusion protein subunit vaccines [44,45]. With regard to polysaccharide conjugate vaccines, it is essential to strike a balance between the cost of developing multivalent vaccines and the coverage of pathogen serotypes in order to maximize their economic impact.

The utilization of GBS surface proteins as carrier proteins to enhance the protective coverage of GBS glycan conjugate vaccines and create multi-protective vaccines has been previously documented in the GBS-related literature [55,57,58,59]. Several GBS surface proteins have been shown to be immunogenic, and among them, alpha C protein, alpha-like protein 3, beta C-protein, ScpB, and GBS80 have been tested as carrier proteins for GBS-conjugate vaccines [21,22,23,40]. Alpha C protein is predominantly present in serotypes Ia, Ib, and II and alpha-like protein 3 is mainly found in serotypes V and VIII, while beta C-protein is primarily found in serotype Ib and GBS80 is mainly present in serotype III and has extremely limited coverage for enhanced protection against GBS infection [22,40]. Only ScpB, as a conserved surface protein, is present in essentially all serotypes examined so far [56,60]. In this study, Sip protein, as an immunogenic protein on the surface of GBS, was also detected in all current GBS serotypes and could be considered as suitable as ScpB for use as a carrier protein for polysaccharide conjugate vaccines from the perspective of cross-protection.

The surface immunogenic protein (Sip) was initially identified and named by Brodeur et al. in 2000 through immunological screening [61]. This 45 kDa surface-localized protein is present in GBS serotypes, Ia, Ib, and II-IX, and the sequence of the *sip* gene is highly conserved in different isolates [60,62]. Animal experiments demonstrated that Sip effectively protected mice from lethal infection with five serotypes of *Streptococcus agalactiae* [61,63]. Martin et al. immunized pregnant mice with recombinant Sip protein and observed that immunity was transferred from mother to offspring, providing protection against a variety of serotypes (75–98% vs. 0–12% in the control group) [49]. Additionally, Shannon et al. found that 99% of 644 pregnant women with clinical natural infection of *Streptococcus agalactiae* produced antibodies to Sip protein in serum, and 97% of 176 healthy born infants contained antibodies to Sip protein in serum, most of which could have immunity for up to six months [64]. These findings suggest that Sip could serve as a potential vaccine target for a GBS polysaccharide conjugate vaccine due to its broad serum coverage, effective antibody protection, maternal immune transmission to offspring, and long-term immune efficacy.

Therefore, in this study, we utilized the CPS of GBS serotype Ia as a model to demonstrate the potential of GBS surface protein Sip as a carrier protein for glycan conjugate vaccines. We first obtained highly pure and structurally correct type Ia CPS of suitable size, and successfully conjugated this CPS with CRM197 and Sip. The antibody titers against Ia polysaccharide antigen in rabbits and mice immunized with Ia-Sip and Ia-CRM197 were not significantly different, but were significantly higher than those in the Ia-polysaccharide group. The results of the opsonophagocytosis and passive immune protection experiments indicated that there was no difference between the Ia-Sip and Ia-CRM197 groups in neutralizing GBS type Ia strains and protecting mice from lethal GBS infection, which was significantly better than that of the Ia-polysaccharide group. Furthermore, serum from the Ia-Sip group had a cross-protective effect on multiple types of GBS strains. The results of challenge experiments in mice demonstrated that the Ia-Sip group could protect mice from lethal doses of the type Ia GBS strain, and there was also cross-protection against the type III strain. These experimental findings further support the potential use of Sip as a carrier protein for GBS glycan conjugate vaccines.

Currently, in the form of polysaccharide conjugate vaccines, the CPS antigen is converted from a T-cell independent antigen to an IgG response that can present epitopes to T cells, trigger cellular immunity, and produce both Th1/Th2 pathways through antibody switching [65,66]. The IgM antibody produced by CPS alone is unable to be transmitted to the fetus, thus providing immune protection only to the mother. In contrast, the polysaccharide conjugate antigen not only stimulates higher levels of IgM antibody production but also induces immune memory and generates higher levels of IgG antibody, which can be transmitted across the placenta to confer maternal immunity on the fetus. On the other hand, our findings also indicate that current polysaccharide vaccines and polysaccharide conjugate vaccines are not effective in stimulating IgA antibody formation (although the latter does produce slightly higher levels of IgA antibodies than the former, overall antibody levels remain low), which aid in acquiring relevant GBS-specific antibodies from maternal milk for defense resist late-onset GBS infection, suggesting a need for alternative methods to stimulate IgA antibody production. For instance, new immunization approaches such as intestinal mucosal immunization could be employed to enhance infant immunity against GBS infection postnatally [67,68].

## 5. Conclusions

In conclusion, our study firstly demonstrated the potential of Sip as a carrier protein for GBS polysaccharide conjugate vaccine, and the polysaccharide conjugate antigen exhibited strong immunogenicity and cross-protection against multiple GBS strains in animal experiments. As a carrier protein, Sip can provide serotype-independent protection and contribute to the coverage of polysaccharide conjugate vaccines against different GBS strains.

## Data Availability

The data presented in this study are available upon reasonable request made to the corresponding author. Correspondence and requests for materials should be addressed to S.C.
